# Invisibility of Racism in the Global Neoliberal Era: Implications for Researching Racism in Healthcare

**DOI:** 10.3389/fsoc.2019.00061

**Published:** 2019-08-14

**Authors:** Beth Maina Ahlberg, Sarah Hamed, Suruchi Thapar-Björkert, Hannah Bradby

**Affiliations:** ^1^Department of Sociology, Uppsala University, Uppsala, Sweden; ^2^Skaraborg Institute for Research and Development, Skövde, Sweden; ^3^Department of Government, Uppsala University, Uppsala, Sweden

**Keywords:** racism, neoliberalism, globalization, migration, healthcare, sociology, inequalities, collaborative research

## Abstract

This paper describes the difficulties of researching racism in healthcare contexts as part of the wider issue of neoliberal reforms in welfare states in the age of global migration. In trying to understand the contradiction of a phenomenon that is historical and strongly felt by individuals and yet widely denied by both institutions and individuals, we consider the current political and socioeconomic context of healthcare provision. Despite decades of legislation against racism, its presence persists in healthcare settings, but data on these experiences is rarely gathered in Europe. National systems of healthcare provision have been subject to neoliberal reforms, where among others, cheaper forms of labor are sought to reduce the cost of producing healthcare, while the availability of services is rationed to contain demand. The restriction both on provision of and access to welfare, including healthcare, is unpopular among national populations. However, the explanations for restricted access to healthcare are assumed to be located outside the national context with immigrants being blamed. Even as migrants are used as a source of cheap labor in healthcare and other welfare sectors, the arrival of immigrants has been held responsible for restricted access to healthcare and welfare in general. One implication of (im)migration being blamed for healthcare restrictions, while racism is held to be a problem of the past, is the silencing of experiences of racism, which has dire consequences for ethnic minority populations. The implications of racism as a form of inequality within healthcare and the circumstances of researching racism in healthcare and its implication for the sociology of health in Sweden are described.

## Introduction

### Racism and Health

Racism is a complex social phenomenon to study, given the variations in historical and geographical contexts as well as non-availability of data especially in the European context which is the main focus in this review. We discuss in this paper the modalities of racism entangled with the enslavement of the African people and colonialism in the context of capitalist development and European modernization. Furthermore, since the focus of the paper is healthcare as part of welfare provision, we follow Paradies et al. (2015) who view racism as an organized system within societies that causes avoidable and unfair inequalities in power, resources, capacities, and opportunities across racial or ethnic groups, which manifests through beliefs, stereotypes, prejudices, or discrimination. Racism can moreover, be internalized, through incorporating racist attitudes, beliefs, and ideologies in one's worldview; this can be interpersonal through interaction between individuals or systemic for example, through lack of control of and access to labor, material, and symbolic resources within a society. This implies that racial inequalities in healthcare can be understood as lack of access to socioeconomic opportunities and resources, within contexts of negative images, stereotypes, prejudice, and discrimination against certain minority groups. It is in this, complex context that racial health disparities described here need to be understood.

Priest and Williams (2018) for example, describe how pervasive global health inequalities exist among what they describe as “stigmatized and disenfranchised ethnic groups (p. 1)” in a wide variety of health conditions including: “earlier onset of illness, increased severity, and progression of disease, higher levels of comorbidity, mortality rates, and poor access to healthcare (p. 1).” Similarly, Arora et al. (2001), show that compared to white populations in the UK, black and minority ethnic populations carry a heavier burden of poor health, premature death and long-term chronic ill health, while refugees, and asylum seekers constitute a high-risk group for mental ill health. Research in Canada suggests that some diseases, including cardiovascular disease, certain cancers, diabetes, and HIV/AIDS, have specific racial/ethnic profiles that disadvantage minority racialized groups (Nestel, 2012). In Sweden, according to Alexander (2009), people who are not ethnically Swedish are more likely to suffer from poor mental and physical health. Hjern (2012) reports that immigrants of non-European background are three to four times more likely than Swedish people to suffer from poor or very poor health. Essén et al. (2000) observe that women with a foreign background, especially those from sub-Saharan Africa, have a higher risk of perinatal mortality than native Swedish women. These perinatal mortality differences could not be explained by existing risk factors and the authors suggest that more intense surveillance should be given to women and newborns from sub-Saharan Africa as a way of reducing perinatal mortality. Racism, as stressed by Priest and Williams and conceptualized by Paradies et al. (2015) is a critical determinant and fundamental cause of inequalities in health, as underlined by Mulinari and Neergaard (2017) in the case of Sweden. Yet, in spite of such disparities, racism, as is becoming clear from a newly initiated research in Sweden by the authors of this review, is difficult to discuss in healthcare encounters (Bradby et al., 2019), largely because it manifests in very subtle ways.

The aim of this review article is therefore to investigate how racism has been rendered invisible in the context of global neoliberalism the political doctrine concerned with how to manage capitalism as an economic system that according to Nkansah-Amankra et al. (2013) shapes social policy, including public health. The role of global neoliberalism is however understood as encompassing other historical events for example, the end of the second world war, the human rights movement in USA and the liberation or anti-colonial movements. Contextualizing racism in healthcare within this broad global neoliberalism will, we argue, enable us to understand, not just how racism is rendered invisible but more significantly, also the marginalization, vulnerabilities and suffering created in the process. This is particularly important because the inability to identify the historical, social, economic, and ideological structuring of racism within social institutions such as healthcare today, seem to result in attributing responsibility for the effects of racism to those individuals and groups who may also be its casualties (Mueller, 2017; Waldman, 2018).

In the section below, we discuss how racism has become unspeakable although nonetheless experienced. We first explore the historical genealogies of racism, after which we examine how neoliberalism has shaped and contributed to the silencing of racism. We conclude the paper by briefly reflecting on the role of academia and advocating for the application of participatory or collaborative research methodologies in order to help create spaces for promoting open discussion of racism in healthcare settings.

## Historical Genealogies and the Shaping of Racism

As a phenomenon entangled with the history of European modernization, racism arguably goes back to the renaissance. According to Earle and Lowe (2005), Europe was already receiving black Africans regularly and in significant numbers ever since the mid fifteenth century, when they were employed as entertainers, dancers, and other categories of workers, but also as slaves, in the cosmopolitan courts. Having black slaves was at the time a sign of social prestige and distinction in these courts. The presence of black people moreover played a necessary counter-image in the construction of European whiteness and civilization (Earle and Lowe, 2005). While being an essential part of European modernization, racism, has in different historical periods assumed different forms including: biblical argument grounded in perceived Christian superiority, biological argument based on science and finally cultural argument (Blaut, 1992; Rogers and Moira, 2005; Cole, 2018).

In its most conspicuous and violent form, racism was manifested in the European capitalist development when large numbers of Africans were shipped to the Americas as slave labor in mines and in sugar, tobacco, and cotton plantations. During this time, public lynching of slaves by slave owners was commonplace (Young, 2005). Direct colonization that entailed, for example, the scramble for Africa during the Berlin Conference in 1884 (Nilsson, 2013), appropriating land and forcibly moving indigenous people to reserves, promoting exploitation, segregation and exclusion of peoples and groups considered inferior or uncivilized then followed. Colonial interventions were justified through a discourse of civilizing mission (Rodney, 1966; Eltis and Richardson, 1997; Baptist, 2014; Salem and Thompson, 2016).

After these conspicuously violent historical moments, but more specifically after the holocaust and genocide during the Second World War, the civil rights movement in the USA, and the liberation or anti-colonial movements in the colonies, racism has seemingly, been forced into obscurity and invisibility (Lentin, 2008).

Following the Second World War and as a response to the Holocaust, UNESCO issued statements in 1951 and 1952, concluding that there are no innate biological differences between different human groups. Race as a biological category was abandoned in science, at least politically (Shapiro, 1952). The focus on race and consequently racism as based on a biological notion meant that racism was viewed as originating in science rather than as a political idea. Therefore, when race was deemed unscientific, racism was consequently seen as done away with: Europe was regarded by some to have gone back to its original nature–as a non-racist society.

As argued by Goldberg (2009), the Holocaust became the “reference point of race” and Europe's colonial history was brushed away and seen as distinct from European identity and modernity. Mulinari and Neergaard (2017) too argue that the focus on the holocaust explicitly obliterates European colonial history, with annihilation as an extreme form of exclusionary racism, whereas exploitative racism still needs black workers. In addition, the shift of the discourse to cultural forms of racism in many European countries has, as argued by Salem and Thompson (2016), managed to obliterate the biological notion of race from the public sphere therefore making it difficult to bring discussion of racism into public arena.

In Sweden, beside the exceptionalism that constructs Sweden as gender equal, anti-racist, and detached from a colonial past, thus rendering race, racism and racializing processes hard to conceptualize, the removal of the term “race” from Swedish law was perhaps the height of racial color-blindness. In 1973, the Swedish government argued to the UN that it was unnecessary to have laws against racism in Sweden as the majority of Swedish people were regarded as anti-racist (Hübinette and Lundström, 2014). Later in 2014, the Integration Minister argued that this removal of race would help Sweden steer away from xenophobia (Carlson, 2011; Rundquist, 2014; Eliassi, 2017; Mulinari and Neergaard, 2017).

Thus, the neoliberal restructuring and silencing of racism has resulted in an ideology of color-blindness which makes racism even more invisible because it removes any suggestion of white supremacy or white guilt from personal thought and public discussion while legitimizing the existing social, political, and economic arrangements which privilege whites. Bonilla-Silva (2014) describes color-blind racism as the ideological cover for a covert and institutionalized system for maintenance of white privilege without identifying those subjected to or those rewarded by that system. At the same time this ideology insinuates that class and culture rather than institutional racism, are responsible for social inequality (Gallagher, 2003). Such a perspective permits white people to define themselves as racially tolerant and claims to adhere to a belief system that does not judge individuals by the “color of their skin.” To others, color-blindness is culturally grounded in an epistemology of ignorance or a process of knowing designed to produce ignorance around white privilege, culpability, and structural white supremacy (Robbins, 2014; Mueller, 2017). From this color-blindness perspective we can understand the difficulties expressed by white Swedish social workers within a study of conceptions of immigrant integration (Eliassi, 2017) in conceptualizing racism in forms other than the way it was enacted in Apartheid South Africa. The social workers identified the cultural identity of immigrants rather than racism as what mainly complicates those immigrants' ability to integrate into the Swedish Society. In the context of color-blind ideology, the focus on cultural and ethnic backgrounds tend to obstruct identification of structural inequalities embedded in society (Mueller, 2017). Since a good deal of research has focused on the rise of neoliberal capitalism (Davis, 2007) which is often seen as signaling the end of racism or instituting a post-racial society (Giroux, 2003) or what Goldberg (2009) defines as evaporating racism, we aim in the next section to highlight the connection between neoliberalism and the silencing of racism.

## Neoliberalism and Racism

Neoliberalism or the doctrine of the free market and related political and individual freedom, was perhaps best articulated by, the Nobel Prize winner economist Milton Friedman (1962) who strongly opposed the type of liberal democracy that developed toward the middle of the nineteenth century, with its emphasis on equality and social welfare which he saw as state intervention and paternalism. What is important in the context of this review paper is however the way Robbins (2014) depicts how contemporary racist practices and structures of inequality are coupled with the authority of neoliberalism, especially in the way that neoliberalism has emptied the social and privatized its expressions. Perhaps most important here is especially the added impact of economic and labor restructuring on different classes within the complex context of color-blindness. The section below examines neoliberalism and related globalization processes, to allow for a better understanding of the structural transformations that may have further muted or silenced racism and the vulnerabilities arising.

### Global Neoliberalism, Decay of the State, and Emerging Vulnerabilities

The focus is to understand how the restructuring through global neoliberalism has, as argued by Dominelli (1999), redefined the welfare state and what implications this has had on labor, welfare, caring, and well-being. Dasgupta (2018) argues that the nation state is declining, and has lost influence over the human condition while national political authority has similarly declined.

The OECD reports (2011, 2015) highlight how even in countries such as Germany, Denmark, and Sweden where the value of egalitarianism has been highly acclaimed, income inequality by class has increased, from the 1980s to today. Meanwhile according to Bergmark (2008); Lundberg and Waldenström (2016), and Therborn (2018), Sweden has the most uneven pattern of wealth distribution in Western Europe. In 2002, for example, the top one percent owned 18% of all household wealth, but by 2017, this had risen to 42% (Therborn, 2018).

One major aspect of the decay of the state is the paradox of loss of control over capital flow while reproduction of racial categories and racialization are ongoing. Even though race and racism may, in the context of color-blindness, be denied, or ignored migrants (a readily racialized category) are nonetheless blamed for the negative impact of these structural transformations on the general population. The loss of control over capital implies there is less possibility for reinvestment or redistribution of wealth in the spaces where that wealth has been produced and this contributes to growing unemployment, poverty, and inequalities. All this is a sign of the break from the previous state of government mastery over national destiny in providing healthcare, education, and welfare. In addition to the decay of the nation state as a result of global neoliberalism, the global south, and particularly the African continent, even in the aftermath of colonialism, remains the supplier of raw material such that profits accumulate for big corporations in the global north (Akokpari, 2001). This resource extraction is well-articulated by Hansen and Jonsson (2014a) in their historical account of European integration where from the 1920s to the 1960s, the central idea and discussion was to create, what was then called, Eurafrica. There was an understanding then that European integration could only succeed through a coordinated exploitation of Africa, which would have entailed European states combining their economic and political capacities. However, the relations between Europe and colonialism have been systematically neglected by European historians and researchers. According to Bhambra (2016), the failure to address the past colonial histories of Europe, leads to the dismissal of the present post-colonialism and multiculturalism or the ways these relationships create vulnerabilities.

The silence around past and present exploitative relations may then explain the way nationalist groups in the global north, reason around migrants being the cause of their problems for taking jobs and welfare meant for the natives (Hansen and Jonsson, 2014b; Schierup and Ålund, 2014; Bhambra, 2016; Therborn, 2018; Dahlstedt and Neergaard, 2019). This is well-articulated by Davidson and Saull (2016) who argue that:

…*.the revival of the far-right is suggestive of the political pathologies in particular and organic necessities of neoliberal political economy. In this context, the far-right plays two roles. On the one hand, it articulates angry resentful grievances across a range of social layers in response to the transformations, instabilities and dislocations of neoliberalism….On the other hand it is an important agent of populist insurgency that has come to provide an important democratic and popular veneer to the transformation wrought by neoliberalism…. (p. 2)*.

As already indicated neoliberalism is a force in the decay of the nation state and democratic welfare (Davidson and Saull, 2016). Moreover, it entails extending the doctrine of the free market to embrace every part of public and personal worlds and entails the transformation of states and governments from being providers of social welfare to promoters of markets and competition. This means, as argued by Monbiot (2017), cutting expenditure on social services including education, healthcare, and other infrastructure; reducing government regulation that can diminish private profits; selling state-owned enterprises, goods, and services to private investors; eliminating the concept of common goods and replacing it with individual responsibility to work hard to succeed in becoming wealthy. This according to Davis (2013), results in a paradox where the poorest people have to find solutions for their health care, education, and social security and, should they fail, they are blamed as lazy.

Trepagnier (2010) articulates this point in relation to policy programs such as affirmative action in USA, aimed at assisting African-Americans and other marginalized minority groups to achieve racial parity. Affirmative action is objected to as unfair gains by blacks, at the expense of whites, which goes against the neoliberal principle that individuals, rather than wider society, should be responsible for their own welfare. Kaika (2017) describes how the doctrine of individual responsibility has affected households around Europe. Many households live in debt, a position that has forced them to re-mortgage their homes in order to pay for increased school or university fees, healthcare or to make up for a reduction in their pensions. In the same breath, the wealthy can exonerate themselves for their hard work, even when they may have inherited their wealth or have had other advantages such as higher education.

At the same time, members of the ruling classes and political parties of both center-left and center-right are, as argued by Davidson and Saull (2016), united in accepting neoliberalism, as the only way of organizing capitalism. This has left the working classes dismantled or fragmented thus also creating a crisis for working class white identity (Robbins, 2014; Mueller, 2017). This crisis is compounded by recruitment of low paid labor including imported migrant labor now increasingly used to offset costs in the provision of welfare as described by Therborn (2018) in the case of Sweden, although the connection between neoliberalism and accentuated vulnerabilities may be unclear to those who are experiencing them. According to Davidson and Saull (2016):

*Individuals may not blame capitalism as a system or on themselves as participants in the system for their personal dissatisfactions, but this does not mean that they have dispensed with the need to find someone or something to blame; but whom?….migration is therefore a central issue here…. neoliberalism contributes to the revival of far-right politics through the global, structural changes that has carried through over the last 40 years (p. 5)*.

While racial humiliation, exclusion, and exploitation of the global south by the global north has continued; the contrast is that the enemy was visibly present during the slave trade and colonial exploitation in the form of a white settler or an expoitative employer in the mines or plantations. Today the enemy is invisible, distributed and remote. Williamson (2017) has similarly noted the shift from an explicitly racial system of stratification for example, based on colonialism, segregation and apartheid, to a system of racial hegemony.

In line with the reasoning above, Therborn (2018) notes how in Sweden “*post-industrial globalized and financialised capitalism has increased economic inequalities by weakening the position of labour, fragmenting the working class and deskilling parts of it through shifts in labour demands (p. 10)*.” Furthermore, the opening of new avenues for capital, through relocation to low wage sites abroad, increases the prospects for extraction of financial rent thus swelling the inequalities. The neoliberal power structures and processes that create these vulnerabilities however remain invisible and as Davidson and Saull (2016) argue, it is part of the reason migrants are seen as the problem in the global north for taking jobs and welfare. Increasing poverty, inequalities, and unemployment arising from the structural adjustment programmes and reforms undoubtedly plays a contributory role to the pressures to migrate for better work opportunities and an associated stream of human trafficking (Butler, 2015; Williamson, 2017).

Migrants and refugees who are able to find employment in the global north are concentrated in the least favorable segments of the labor market including less qualified positions, which the natives may not want to take (Wolgast et al., 2018). With cutbacks in public and private sectors that have arisen from neoliberalist austerity labor intensification and exhaustion for the remaining work force has resulted in all sectors (Green, 2004). This process may be more intensively experienced in the healthcare sector where labor is by definition proximate and cannot be out-sourced, as discussed in the following section focusing on the case of migration of nurses from the global south, and their experiences of racism.

### Migrant Labor and Racism: The Case of BLACK African Nurse Migrants

Taking nursing as an example, the paradox arising from neoliberal reforms is work intensification and related, exhaustion, stress, and work related ill-health as articulated by Selberg (2013), concerning nurses in Sweden. The resulting shortage of nursing staff for example, in the UK but also in other EU countries, has resulted in hospitals, but also private agencies, recruiting nurses from Eastern European countries, and from the global south (Hagey et al., 2001; McElmurry et al., 2006; Batnitzky and McDowell, 2011; Kaelin, 2011; Germack et al., 2015). Apart from the ethical concerns (McElmurry et al., 2006; Bradby, 2014) about removing health personnel from poor countries, nurse migrants especially black nurses, are also placed in vulnerable, inequitable work roles, sites, shifts, and days unattractive to other nurses. What Kaelin (2011) calls “care drain,” is a double bind for those migrating to escape the structural violence of poverty, joblessness, and inequalities in source countries, only to experience continued structural violence through institutional racial discrimination and mistreatment in destination countries. According to Likupe and Archibong (2013), the black African nurses experience racially motivated discrimination from employers and colleagues but also note that:

*On the one hand, Black African nurses are migrants who are recruited to a subordinate position at the bottom of the nursing hierarchy…. they are often better educated and experienced than their British colleagues ….Managers seem to have legitimated nursing assistants' feeling of superiority by assigning them to supervise Black African nurses (p. 241)*.

Besides being denied career development opportunities, Black African nurses are, not believed, or trusted which leads to stress and poor health. Estacio and Saidy-Khan (2014) for example, report racial micro-aggressions experienced by migrant nurses in two hospitals in the midlands of England, through bullying at the hands of their nurse colleagues and racial preferences by patients as well as institutional racism that hinder further training and promotion of the migrant nurses. This led to feelings of anger, frustration and even paranoia. In spite of these experiences, migrant nurses were found to also downplay the incidents as trivial because of their vague and subtle nature. The study authors add:

*Contemporary forms of racism are difficult to challenge because quite often its perpetrators are not aware that their actions can be considered as racist. New forms of racism can be problematic because these reflect deep-seated prejudices that can be difficult to overcome (*Estacio and Saidy-Khan, 2014, *p. 1)*.

The fact that both the perpetrators as well as those to whom racism is directed have difficulties recognizing racism, calls for critical reflection on how to research racism in healthcare. Being seen as a product of the past and in the context where a major impact of global neoliberalism has been silencing racism, we argue that racism in healthcare becomes difficult to discuss and is silenced (Bradby et al., 2019).

In addition, the idea that racism or the refuted racial differences could be part of the medical profession is disturbing as argued by Hoberman (2012), since racism is considered as a violation of the core ethics of a profession that is supposedly based on caring, equality, and solidarity. Healthcare providers have strong autonomy and professional identity when compared to other professionals in the welfare system, and usually see themselves as defenders of rationality. The unequal treatment of patients or of staff on racial grounds would appear to be a grave violation of medical ethics and would, for the most part, not be expressed publically and explicitly (Bradby, 2010) which makes it even more difficult to discuss. The question then is how to change this complex phenomenon that is entangled with history, neoliberalism and color-blind ideology which enables whites to ignore the degree to which race is, entangled with unequal power relations and is a force for exclusion and discrimination (Omi, 2001; Giroux, 2003). How can this situation be changed and what role might research within academic institutions play in making such changes? We go into more detail on this in the next section.

It is thus equally important to examine how academia has been involved in the study of race and racism. According to Mulinari and Neergaard (2017), except for the newly emerging critical racial theory, the study of racism and racial inequalities has been extremely marginal in Sociology in Sweden, and the Scandinavian countries in general. Given that one aim in our newly initiated research (Bradby et al., 2019) is to eventually find ways of promoting open discussion on the experience and effects of racism, it is therefore important to understand how academia and more specifically sociology has engaged with race and racism.

## Innovating With Participatory or Collaborative Approaches in Researching Racism in Healthcare

Before discussing participatory approaches to research, we briefly reflect on the role of academia and of sociology in particular. The focus of this review has been on the difficulties of researching racism within the history of political and economic restructuring of societies, states, and governments because in the context of globalized neoliberalism racism has been silenced. Such a focus can be seen to situate the paper in post-colonial studies and literature and as argued by Go (2013a), thus also on colonial domination. However, because post-colonial studies mainly focus on the global south, it is important to have a reflective stance on the role of social sciences particularly, sociology and its sister discipline anthropology, in an attempt to understand how they have or have not engaged with the subject of race and racism. Here the ideas articulated by Bhambra (2014), are in line with Mulinari and Neergaard's (2017) suggestion that the study of racism and racial inequalities has been extremely marginal in sociology in Sweden. According to Bhambra (2014), sociology has mainly focused on modernity as a European production distinct from its colonial entanglement and states:

*The sociological understanding of modernity typically rests on ideas of the modern world emerging out of the processes of economic revolution located in Europe and underpinned by the cultural changes by the Renaissance, Reformation, and Scientific revolution ….Coterminous with this argument is the idea that the rest of the world was external to these historical processes and that colonial connections and processes were insignificant to their development (p. 653)*.

This means that sociology has not addressed colonial relationships as integral to modernity (Lowe, 2015), with a suppression of colonial and imperial dynamics in classical sociological texts. According to Go (2013a), post-colonial theory has had more influence in the humanities, and little in sociology, although Bourdieu in his earlier work is shown to have recognized racism as a racialized system of domination and force in restructuring social relation. Elsewhere, Go (2013b) has emphasized the need for sociology to focus on the interactional constitution of social units (colonizers and colonized), processes and practices across space. Such a relational focus would mean, as argued by Hansen and Jonsson (2014a), that the discipline would need to pay more attention to the history and politics of the knowledge produced and the ways in which the practice and teaching of sociology could engender the epistemologies of empire. In contrast, anthropology, as Levi-Strauss (1966) articulates: “is the science of culture as seen from the outside (p. 126).” Lewis (1973) describes the role of the anthropologist during the colonial time in the following way:

*Since the anthropologist worked amid the profound economic and political changes which accompanied the confrontation between the West and the rest of the world, he was often called upon to provide information and advice to the West in its efforts to manipulate and control the non-western world. He provided the information either directly or indirectly and became, thereby, implicated in the process of colonization (p*. 582).

Another area of concern is the declining focus on race studies in academia and when issues of race and racism are studied, the methods and reporting tend to down-play racialized inequality. Outdated questions about racial tolerance have been used to demonstrate “progress” in attitudes, while other research locates problems in minority culture rather than structural inequalities (Bonilla-Silva, 1997; Bonilla-Silva and Baiocchi, 2001). The way sociologists report their results thus tend to distort or minimize the structural features particularly in the context of the neoliberal doctrine in restructuring of the economy and the ideology of color-blindness discussed earlier. In development studies there has been very little conceptualization of race (White, 2002; Wilson, 2012), with much more focus on challenging eurocentrism (Bonilla-Silva, 1997). Kothari (2006) has wondered whether, the invisibility around “race” is because it is considered relatively unimportant in shaping inequalities, injustice and poverty. This question is critical because, at the same time as race as a category of power and social differentiation is invisible, ignored, and denied, other categories such as class, gender, and ethnicity are much discussed, not to mention the increased focus on insecurity and terrorism. Moreover, development aid is framed within a relationship between aid donors and aid recipients as part of the binary of developed/underdeveloped. Understanding the historical relationship between developed donors and undeveloped recipients implies a range of global distinctions, hierarchies, and relationships that intersect with other categories such as class, gender, and ethnicity as they become pertinent to race and racism. Just as post-colonial theory omits race, sociology too, as already pointed out, has not engaged much with the empire and colonial domination around which race and racism developed. It is in this context that Williamson's (2017) conclusion, that future research should have a critical globalization perspective, makes sense.

Given the complexities described in the sections above, it is clear there is dire need to use research methodologies that promote critical reflection and open discussion on what is going on, not just for the researchers but also for the researched. This is largely why we have proposed the use of participatory or collaborative processes in our research, to allow for engagement of a broad spectrum of stakeholders. The attempt is to create communicative spaces that would lead to dialogue and critical reflection around the issue of race and racism, which would also indicate the policy action, change and anti-racist practice. Moreover, given that the perpetrators and recipients may not recognize racism during their interaction in a healthcare setting, perhaps one other method could entail asking black minority immigrant nurses and other health professionals to keep diaries of their interaction with colleagues and patients as basis for further, collective reflection.

Creating an open discussion around racism or identifying a language that enables addressing and understanding racism within healthcare settings may, as already indicated above, require among other approaches, the use of participatory or collaborative research processes. Such a process allows the application of the theoretical perspective of communicative action and related opening of communicative spaces, first developed by the critical theorist Habermas (1984) but later articulated by participatory action researchers (Kemmis and McTaggart, 2005; Kemmis, 2006; Godin et al., 2007; Gaya and Reason, 2009), relevant for understanding communication about complex but contested issues between unequal partners. Despite inherent ambiguities in Habermas's conceptualizations (see Honneth and Joas, 1991), communicative action allows participants, to consciously and deliberately reach intersubjective agreement, as the basis for mutual understanding about what to do in their particular practical situation, in this case how to enhance discussion on racism that informs action to reduce the damage of racism in healthcare interactions. What we are suggesting in others words is a research process that engages different stakeholders as active co-researchers in order to allow co-production of knowledge that leads to action and change.

To enhance communicative action and create space for dialogue on racism or even to frame the research problem, there is need to understand the historical events, social and economic structural processes that may have muted the collective ability to speak about racism. This review paper hopefully accomplishes that goal.

## Conclusions

We have attempted to review how and why racism remains largely invisible and hugely underestimated, although it is a historical phenomenon well-ingrained in modernity and still remains a clear category of power structuring and discrimination. In spite of being a critical determinant and fundamental cause of health inequalities, racism is difficult to discuss in healthcare encounters, which implies an urgent need for more research to explore ways of creating and promoting an open discussion contributing to constructive change in healthcare settings.

The development of global neoliberalism has played a major role in silencing and making racism invisible. Apart from the decay of the nation state, as provider of welfare, the neoliberal reforms and restructuring have led to a shift of the national wealth to the elite class. This in turn has led to the paradox of the loss of statutory or public control over capital flow, while reproduction of race categories and racialization are ongoing. The lack of explicit discussion of race and racism means that individual and organizational discrimination is hard to name or challenge, even as they are opened up for exploitation as cheap labor. Privatization of the public space has disconnected power from social obligation, making it more difficult for individuals and institutions to have a language that can accommodate the principles of racial justice as a common good. In addition, the race color-blind ideology enables powerful white groups to ignore the degree to which racism is entangled with unequal power relations and as a force for exclusion and discrimination. It is in this context of silence and obscurity that academia is called upon to revisit modernity and its relation to empire with a critical perspective on globalization.

## Author Contributions

All authors listed have made a substantial, direct and intellectual contribution to the work, and approved it for publication.

### Conflict of Interest Statement

The authors declare that the research was conducted in the absence of any commercial or financial relationships that could be construed as a potential conflict of interest.
